# Regional mapping of species‐level continuous foliar cover: beyond categorical vegetation mapping

**DOI:** 10.1002/eap.2081

**Published:** 2020-02-24

**Authors:** Timm W. Nawrocki, Matthew L. Carlson, Jeanne L. D. Osnas, E. Jamie Trammell, Frank D. W. Witmer

**Affiliations:** ^1^ Alaska Center for Conservation Science University of Alaska Anchorage 3211 Providence Drive Anchorage Alaska 99508 USA; ^2^ Department of Biological Sciences and Alaska Center for Conservation Science University of Alaska Anchorage 3211 Providence Drive Anchorage Alaska 99508 USA; ^3^ Department of Environmental Science & Policy Southern Oregon University 1250 Siskiyou Blvd. Ashland Oregon 97520 USA; ^4^ Department of Computer Science & Engineering University of Alaska Anchorage 3211 Providence Drive Anchorage Alaska 99508 USA

**Keywords:** Alaska, Arctic, Bayesian statistical learning, big data, foliar cover, gradient boosting, North Slope, proportional abundance, remote sensing, species distribution, vegetation map

## Abstract

The ability to quantify spatial patterns and detect change in terrestrial vegetation across large landscapes depends on linking ground‐based measurements of vegetation to remotely sensed data. Unlike non‐overlapping categorical vegetation types (i.e., typical vegetation and land cover maps), species‐level gradients of foliar cover are consistent with the ecological theories of individualistic response of species and niche space. We collected foliar cover data for vascular plant, bryophyte, and lichen species and 17 environmental variables in the Arctic Coastal Plain and Brooks Foothills of Alaska from 2012 to 2017. We integrated these data into a standardized database with 13 additional vegetation survey and monitoring data sets in northern Alaska collected from 1998 to 2017. To map the patterns of foliar cover for six dominant and widespread vascular plant species in arctic Alaska, we statistically associated ground‐based measurements of species distribution and abundance to environmental and multi‐season spectral covariates using a Bayesian statistical learning approach. For five of the six modeled species, our models predicted 36% to 65% of the observed species‐level variation in foliar cover. Overall, our continuous foliar cover maps predicted more of the observed spatial heterogeneity in species distribution and abundance than an existing categorical vegetation map. Mapping continuous foliar cover at the species level also revealed ecological patterns obscured by aggregation in existing plant functional type approaches. Species‐level analysis of vegetation patterns enables quantifying and monitoring landscape‐level changes in species, vegetation communities, and wildlife habitat independently of subjective categorical vegetation types and facilitates integrating spatial patterns across multiple ecological scales. The novel species‐level foliar cover mapping approach described here provides spatial information about the functional role of plant species in vegetation communities and wildlife habitat that are not available in categorical vegetation maps or quantitative maps of broadly defined vegetation aggregates.

## Introduction

The ability to understand ecological processes at landscape scales depends on representations of vegetation that quantitatively reflect observed patterns in plant communities through space and time (Cushman 2010, Cushman et al. 2010c). Continuous spatial representations of fundamental ecological units, such as habitat resources or individual species, catalyze hypothesis‐driven analyses of ecological drivers and responses across ecological and spatial scales and enable accurate change assessments (Cushman et al. 2010b, Lausch et al. 2015, Coops and Wulder 2019). Improved understanding of the interactions between ecological drivers and responses across scales provides insight into fundamental problems in ecology and can contribute to better management of natural resources. Spatially explicit and quantitative understanding of vegetation patterns is necessary to distinguish the effects of climate change, development, and management actions from existing spatial heterogeneity.

Vegetation responses to a changing climate are widespread in the Arctic: shifts in the distribution, foliar cover, heights, and phenology of numerous plant species have been associated with increasing temperature, growing season length, and active layer depth (e.g., Tape et al. 2006, Pearson et al. 2013, Fraser et al. 2014). Increases in temperature do not affect all plant species similarly; communities are often restructured rather than merely shifted spatially (Cushman et al. 2010a). In turn, shifts in cover and height of plant species driven by environmental changes cause feedbacks that alter environmental conditions (e.g., active layer temperature in Frost et al. 2018). Shifts in the characteristics of plant communities also drive herbivore responses (Boelman et al. 2015, Tape et al. 2016), causing feedback cycles whereby the activities of herbivores interact with environmental conditions to influence the responses of plant communities (e.g., Christie and Ruess 2015). Quantitative analyses of the complex interactions and feedbacks among plants, soils, climate, and herbivores at the landscape scale require quantitative spatial representations of distribution, abundance, and trends for ecologically meaningful units of vegetation, such as the species or community.

Qualitative and subjective categorical vegetation maps, which are currently widely used, pose substantial limitations in our ability to quantify species‐ or community‐level distribution, abundance, trends, or relationships with ecological drivers across landscapes (Cushman et al. 2010c, Coops and Wulder 2019). Non‐overlapping grids of discrete, subjectively defined vegetation types (i.e., categorical vegetation and land cover maps) depend on several assumptions: (1) individual species form deterministic clusters of multiple but finite discrete vegetation types; (2) quantitative variation within vegetation types is not important or does not exist; and (3) transitions between vegetation types are always abrupt discontinuities (Evans and Cushman 2009, Cushman et al. 2010c). While it is possible to serially discretize the cover or abundance of individual plant species within these subjective vegetation types, doing so can only be accomplished as a mean and variance per type for each species. Furthermore, while non‐overlapping discrete types may represent the ecological pattern of one to several species reasonably well, they often fail to do so for numerous other species (Cushman et al. 2010c). Vegetation analyses based on the assumptions described above are not consistent with theories of individualistic response of species and niche (Schmidtlein and Sassin 2004, Evans and Cushman 2009, Feilhauer et al. 2011, Harris et al. 2015) because they subjectively subsume broad ranges of multi‐species response variation and imply abrupt environmental discontinuities where generally none exist (Evans and Cushman 2009, Cushman et al. 2010c, Lausch et al. 2015).

Ecological communities are not predetermined or static clusters, but instead they are dynamically arranged from the continuous responses of individual species to spatially and temporally variable environmental and biotic gradients. The responses of individual species to environmental and biotic gradients are recognized to be the primary drivers of variation in vegetation communities (Gleason 1926, Whittaker 1967, Lortie et al. 2004, Cushman et al. 2010a,c). These individual species responses to environmental and biotic gradients form continuums in niche space that result in species patterns in physical space (Grinnell 1928, Hutchinson 1957, Colwell and Rangel 2009). Communities are formed by the establishment, survival, and reproduction of individuals of constituent species based on tolerance to environmental conditions, interactions among species with geographic access to the same physical space, and stochastic events. The species is therefore the fundamental scalable unit by which environmental and biotic gradients, along with biohistorical factors, stochastic events, and dispersal limitations, drive vegetation patterns across landscapes (Cushman et al. 2010a).

A continuous gradient data model enables a niche‐based approach to vegetation mapping such that species‐level variation is captured in the resulting spatial representation (Cushman et al. 2010a,c). Gradient representations of landscape pattern realistically represent continuous natural variation with minimal assumptions for both organisms and processes (McGarigal and Cushman 2005, Cushman et al. 2010c). Unlike categorical vegetation maps, niche‐based quantitative gradient maps avoid the implicit assumptions that landscapes are divided by abrupt discontinuities and that plant communities are determinate (Evans and Cushman 2009, Cushman et al. 2010a,c, Feilhauer et al. 2011, Harris et al. 2015). The niche‐based gradient mapping approach preserves the continuous nature of observed variation and thus is compatible with ordination methods for analyzing community composition, which are also founded on the continuum concept of individualistic response of species (Feilhauer et al. 2011). The gradient data model integrates vegetation responses across spatial and ecological scales (Cushman et al. 2010b, Harris et al. 2015), avoiding the need to tailor discrete classifications to unique scales. Despite the theoretical benefits of applying the niche‐based gradient approach to vegetation mapping, it has only been applied in a few spatial analyses of vegetation, primarily in the context of mapping floristic gradients derived from ordination in small geographic areas (Schmidtlein and Sassin 2004, Feilhauer et al. 2011, Harris et al. 2015).

In Arctic Alaska, both categorical and gradient paradigms have been applied to map vegetation patterns, but a niche‐based gradient approach is absent. Spatial representations of vegetation in northern Alaska have been dominated by subjectively defined categorical approaches (e.g., Markon 1986, Jorgenson et al. 1994, 2009a,b, Markon and Derksen 1994, Boggs et al. 1999, Muller et al. 1999, Jorgenson and Heiner 2003, Ducks Unlimited 2013). More recently, arctic Alaska researchers have quantified gradient patterns for foliar cover, height, and biomass of vegetation using plant functional types or other broad categories, including vegetation as a whole (e.g., Beck et al. 2011, Langford et al. 2016, Macander et al. 2017, Berner et al. 2018). These efforts have been successful at mapping the spatial variation of functional vegetation categories and are useful for quantifying biophysical patterns of vegetation across landscapes with applications where the plant functional type or broad category is relevant (e.g., carbon sequestration, fuels for fire, and greening of the Arctic). Neither the existing categorical maps nor the existing gradient maps quantify the distribution and abundance responses of species to environmental and biotic gradients (i.e., the realized niche). Therefore, they do not allow exploration of relationships among plant species, environmental gradients, wildlife, soils, and ecological processes. Here, we demonstrate a novel approach to quantifying continuous landscape vegetation patterns at the species level using a test case in arctic Alaska. Our approach is an alternative to categorical vegetation mapping and addresses the recent calls by Cushman et al. (2010c) and Coops and Wulder (2019) to avoid discrete classifications.

## Methods

Three components were required to quantify species‐level spatial patterns of foliar cover: (1) reliable observations of species distribution and foliar cover at a relevant spatial resolution; (2) consistent measurements of covariates that represented the existing environmental and biotic gradients; and (3) a statistical learning method capable of predicting complex patterns of continuous responses from numerous, potentially weak correlations in relatively small samples.

We use the term “covariate” in this paper to refer to an individual measured property, as this term is more familiar to ecologists; the term “feature” is typically used in statistical learning literature. We conducted spatial processing in ArcGIS Pro 2.2.3 (ESRI, Redlands, California, USA) with Python 3.5.3; topographic calculations in Geomorphometry and Gradient Toolbox 2.0 (Cushman et al. 2010c, Evans et al. 2014); stream network delineation in TauDEM 5.3.7 (Tarboton and Baker 2008); statistical modeling in the Anaconda 2019.10 distribution of Python 3.7.4 with XGBoost 0.90 (Chen and Guestrin 2016), Scikit‐learn 0.21.3 (Pedregosa et al. 2011), GPy 1.9.9 (GPy 2014), and GPyOpt 1.2.5 (González et al. 2014); and prediction post‐processing using R 3.6.1 (R Core Team [Link]) and RStudio Server 1.2.5019 with sp 1.3‐2 (Pebesma and Bivand 2005), raster 3.0‐7 (Hijmans 2017), and rgdal 1.4‐7 (Bivand et al. 2018). Code for all analyses referenced in this study is available as a git repository (Nawrocki 2019a).

### Vegetation field data collection

We sampled 185 stratified‐random sites in an extensive series of grids in the Arctic Coastal Plain and Brooks Foothills of Alaska (as defined by Nowacki et al. 2001) from July 2012 to July 2017. At each site, we sampled vegetation foliar cover in a 30 m radius plot consisting of three 25‐m transect lines at 120° intervals. Lines originated 5 m from the plot centroid and radiated out toward the perimeter. We quantitatively measured foliar cover as the sum of unique species intersections in any canopy layer with an approximately 1 mm radius laser at 50 points along each of the three transect lines divided by the total possible number of unique intersections per plot (Herrick et al. 2017). This estimate of foliar cover is expressed as the percentage of the total measured ground area covered by live or rooted dead plant material per plot. Our cover calculation method is equivalent to the any‐hit cover described by Karl et al. (2017). We integrated these data into a standardized database (Nawrocki 2019b) with vegetation observations from thirteen additional survey and monitoring data sets collected in northern Alaska from 1998 to 2017 for a total of 2,872 plots (Table 1).

**Table 1:** Extensive, ground‐based vegetation survey data sets with distribution and foliar cover data for northern Alaska collected between 1998 and 2017 that we integrated into a standardized database

Project (data reference)	Funder, originator	Years	Methods	Plot size (m)	Number
NPR‐A Assessment, Inventory, and Monitoring (this study)	BLM, ACCS	2012–2017	quantitative line‐point intercept	30 radius	185
Colville Small Mammal Surveys 2015 (Flagstad and Nawrocki, *Unpublished data*)	ADF&G, ACCS	2015	semi‐quantitative visual estimate	10 × 10	16
Ecological Land Survey of the NPS Arctic Network (Jorgenson et al. 2009a)	NPS, ABR	2002–2008	semi‐quantitative visual estimate	5–30 radius, 5 × 10, 10 × 10	807
Ecological Land Survey of the Selawik National Wildlife Refuge (Jorgenson et al. 2009b)	USFWS, ABR	2007–2008	semi‐quantitative visual estimate, quantitative grid‐point intercept	10 radius, 10 × 10, (plus others)	271
Balsam Poplar (*Populus balsamifera* L.) Communities on the Arctic Slope of Alaska (Breen 2014)	Amy Breen (UAF)	2003–2006	Braun‐Blanquet visual estimate	10 × 10	29
Gates of the Arctic National Park Land Cover Mapping (Boggs et al. 1999)	NPS, ACCS	1998	semi‐quantitative visual estimate	10 × 10	108
North Slope Land Cover Mapping (Ducks Unlimited 2013)	NSSI, ACCS	2008–2011	semi‐quantitative visual estimate	10 × 10, (plus others)	146
Plant Associations in Yukon‐Charley Rivers National Preserve (Boggs and Sturdy 2005)	NPS, ACCS	2003	semi‐quantitative visual estimate	10 × 10	66
Fortymile River Region Assessment, Inventory, and Monitoring (Boucher et al. *Unpublished data*)	BLM, ACCS	2016–2017	quantitative line‐point intercept	20 × 80–25 × 100	3
Vegetation Monitoring in Selawik National Wildlife Refuge (Jorgenson et al. 2009b)	USFWS	2005	Braun‐Blanquet visual estimate	variable	155
Regional Cover Mapping of Tundra Plant Functional Types (Macander et al. 2017)	Shell, ABR	2012	quantitative line‐point intercept	55 radius	106
Shell Onshore/Nearshore Environmental Studies (Murphy et al. 2016)	Shell, ABR	2010–2012	semi‐quantitative visual estimate	10 radius, (plus others)	711
Selawik National Wildlife Refuge Vegetation Mapping Surveys (Jorgenson et al. 2009b)	USFWS	1996–1998	semi‐quantitative visual estimate	variable	96
Vegetation Monitoring in Interior Alaska National Wildlife Refuges (Lieberman et al. *Unpublished data*)	USFWS	2013–2014	semi‐quantitative visual estimate	15 radius, 5 × 5–30 × 30	173

To ensure an adequate number of samples for the cross‐validation train and test partitions of our statistical analyses, we mapped the vascular plant species that we observed with foliar cover ≥ 1% in at least 80 of our 185 plots. *Carex aquatilis*,* Eriophorum angustifolium*,* Eriophorum vaginatum*,* Rhododendron tomentosum*,* Salix pulchra*, and *Vaccinium vitis‐idaea* fit our selection criteria. Genus names are abbreviated hereafter for these species for convenience. The six selected species are associated with dominant vegetation communities in arctic Alaska and are linked to major ecological processes, such as soil ice dynamics, tussock formation, and snow retention (Table 2).

**Table 2:** Selected vascular plant species representing dominant vegetation patterns in Arctic Alaska relative to prevalence in our vegetation observation data collected from 2012 to 2017, discrete vegetation types in the North Slope Land Cover map (Ducks Unlimited 2013), and ecosystem processes

Species	Prevalence in our data (%)	Land cover classes with potential for ≥ 25% foliar cover of target species	Linkage to ecosystem processes
*Carex aquatilis*	58	freshwater marsh *Arctophila fulva*, freshwater marsh *Carex aquatilis*, low‐tall willow, mesic sedge‐dwarf shrub, tidal marsh, wet sedge, wet sedge‐*Sphagnum*	wetland indicator, soil ice dynamics (troughs, low‐centered polygons, drained thaw lakes, etc.), wildlife habitat and forage
*Eriophorium angustifolium*	45	freshwater marsh *Carex aquatilis*, low‐tall willow, mesic sedge‐dwarf shrub, wet sedge‐*Sphagnum*, wet sedge	wetland indicator, soil ice dynamics (troughs, low‐centered polygons, drained thaw lakes, etc.), wildlife habitat and forage
*Eriophorium vaginatum*	45	alder, tussock shrub tundra, tussock tundra	tussock formation, soil ice dynamics (high‐ and flat‐centered polygons), nutrient cycling, wildlife habitat and forage (e.g., preferred late spring forage for caribou and ptarmigan)
*Rhododendron tomentosum*	44	dwarf shrub, tussock shrub tundra, tussock tundra, mesic sedge‐dwarf shrub	associational herbivore resistance, ethnobotanical uses, post‐fire succession
*Salix pulchra*	46	low‐tall willow, tussock shrub tundra	shrub expansion, snow retention, hydrography, wildlife habitat and forage (e.g., preferred late spring and summer forage for caribou and ptarmigan)
*Vaccinium vitis‐idaea*	45	dwarf shrub	subsistence, wildlife habitat and forage (e.g., for voles, lemmings, sparrows, bears, and caribou in winter)

### Environmental covariates

We selected a suite of 18 environmental covariates to represent climatic, topographic, and hydrographic conditions across the modeling area. We created two‐decade averages for each climate metric using data from Scenarios Network for Alaska and Arctic Planning (SNAP 2018) to better match the 20‐yr time interval of our integrated vegetation observation data. A historic decadal average (Climate Research Unit Time Series 3.1) represented years 2000 to 2009, while a projected decadal average (Representative Concentration Pathway 6.0) represented years 2010 to 2019. Topographic covariates included or were calculated from the USGS 3D Elevation Program (3DEP) 2 arc‐second (approximately 60 m) Digital Elevation Model (DEM). We selected the 2 arc‐second DEM because it was the finest available resolution of elevation data with continuous coverage over our area of interest at the time of our analyses. Because of the moderate resolution of the 2 arc‐second DEM, our calculated topographic and hydrographic metrics provided a coarse approximation of topography and hydrography relative to the resolution of our field sampling. We derived stream distance metrics from a stream network with stream order calculated from the 2 arc‐second DEM using an accumulation threshold of 50 cells. Additionally, we estimated distance from floodplain by combining the floodplains delineated in the Landscape‐level Ecological Mapping of Northern Alaska (Jorgenson and Grunblatt 2013) and The Alaska Yukon Region of the Circumboreal Vegetation Map (Jorgenson and Meidinger 2015) with a buffered stream network where buffer distance (*d*) varied as an arbitrary function of stream order (*n*): *d* = 10*n* ^2^. The suite of 18 environmental covariates (Table 3) represented the major temperature, moisture, topographic, and hydrographic gradients likely to influence vegetation patterns.

**Table 3:** Environmental covariates with available spatially explicit data appropriate to a 30 × 30 m resolution and corresponding source data sets and metric calculation references

Covariate	Source data	Data source/Methods reference
Date of freeze 2000–2019	CRU3.1 and RCP 6.0	SNAP (2018)
Date of thaw 2000–2019	CRU3.1 and RCP 6.0	SNAP (2018)
Growing season length 2000–2019	CRU3.1 and RCP 6.0	SNAP (2018)
Summer warmth index 2000–2019	CRU3.1 and RCP 6.0	SNAP (2018)
Total annual precipitation 2000–2019	CRU3.1 and RCP 6.0	SNAP (2018)
Linear aspect	USGS 3DEP 2 Arc‐second DEM	Evans et al. (2014)
Compound topographic index	USGS 3DEP 2 Arc‐second DEM	Moore et al. (1993), Gessler et al. (1995)
Elevation	USGS 3DEP 2 Arc‐second DEM	USGS
Heat load index	USGS 3DEP 2 Arc‐second DEM	McCune and Keon (2002)
Integrated moisture index	USGS 3DEP 2 Arc‐second DEM	Evans et al. (2014)
Roughness	USGS 3DEP 2 Arc‐second DEM	Blaszczynski (1997), Riley et al. (1999)
Site exposure	USGS 3DEP 2 Arc‐second DEM	Evans et al. (2014)
Slope	USGS 3DEP 2 Arc‐second DEM	Evans et al. (2014)
Surface area ratio	USGS 3DEP 2 Arc‐second DEM	Evans et al. (2014)
Surface relief ratio	USGS 3DEP 2 Arc‐second DEM	Pike and Wilson (1971)
Distance to large streams (orders 3–9)	USGS 3DEP 2 Arc‐second DEM	Tarboton and Baker (2008)
Distance to small streams (orders 1–2)	USGS 3DEP 2 Arc‐second DEM	Tarboton and Baker (2008)
Distance to floodplains	USGS 3DEP 2 Arc‐second DEM, Landscape Level Ecological Mapping of Northern Alaska, Circumboreal Vegetation Map – Alaska and Yukon	Tarboton and Baker (2008), Jorgenson and Grunblatt (2013), Jorgenson and Meidinger (2015)

### Spectral covariates

Spectral gradients indirectly measure environmental and biotic gradients (Walker et al. 2003, Schmidtlein and Sassin 2004, Raynolds et al. 2008, Feilhauer et al. 2011, Harris et al. 2015) in addition to multi‐canopy layer reflectance from plant physical structures (Karl et al. 2017). Our statistical models thus inferred otherwise unrepresented environmental and biotic gradients from remotely sensed multi‐season spectral data. We calculated cloud‐reduced, maximum NDVI composites in Google Earth Engine (see Gorelick et al. 2017) for Landsat 8 bands 1–7 plus Enhanced Vegetation Index‐2 (EVI2), Normalized Burn Ratio (NBR), Normalized Difference Moisture Index (NDMI), Normalized Difference Snow Index (NDSI), Normalized Difference Vegetation Index (NDVI), and Normalized Difference Water Index (NDWI). We generated maximum NDVI composites from the Top‐Of‐Atmosphere (TOA) reflectance image collection filtered to the months of May, June, July, August, and September from 2013 through 2017 (Table 4; see Chander et al. 2009 for a description of the TOA reflectance method). We did not perform any additional atmospheric corrections to TOA reflectances but did impute missing data using nearest neighbors where clouds obscured all available images for the month. We included multi‐season rather than just midsummer spectral covariates because Langford et al. (2016) and Macander et al. (2017) found improved model performance with inclusion of multi‐season properties for mapping of tundra plant functional types.

**Table 4:** Methods references for spectral bands (Landsat 8) and calculation of metrics

Covariate (May–September)	Processing equation	Reference
Band 1: Ultrablue (UB)	na	Barsi et al. (2014)
Band 2: Blue (BLU)	na	Barsi et al. (2014)
Band 3: Green (GRE)	na	Barsi et al. (2014)
Band 4: Red (RED)	na	Barsi et al. (2014)
Band 5: Near Infrared (NI)	na	Barsi et al. (2014)
Band 6: Shortwave Infrared 1 (SI1)	na	Barsi et al. (2014)
Band 7: Shortwave Infrared 2 (SI2)	na	Barsi et al. (2014)
Metric 1: Enhanced Vegetation Index‐2 (EVI2)	(RED − GRE)/(RED + (2.4 × GRE) + 1)	Jiang et al. (2008)
Metric 2: Normalized Burn Ratio (NBR)	(NI − SI2)/(NI + SI2)	Key and Benson (1999)
Metric 3: Normalized Difference Moisture Index (NDMI)	(NI − SI1)/(NI + SI1)	Jin and Sader (2005)
Metric 4: Normalized Difference Snow Index (NDSI)	(GRE − SI1)/(GRE + SI1)	Hall et al. (1995)
Metric 5: Normalized Difference Vegetation Index (NDVI)	(NI − RED)/(NI + RED)	Tucker (1979)
Metric 6: Normalized Difference Water Index (NDWI)	(GRE − NI)/(GRE + NI)	Gao (1996)

Note

na, not applicable.

### Study area

**Figure 1:**
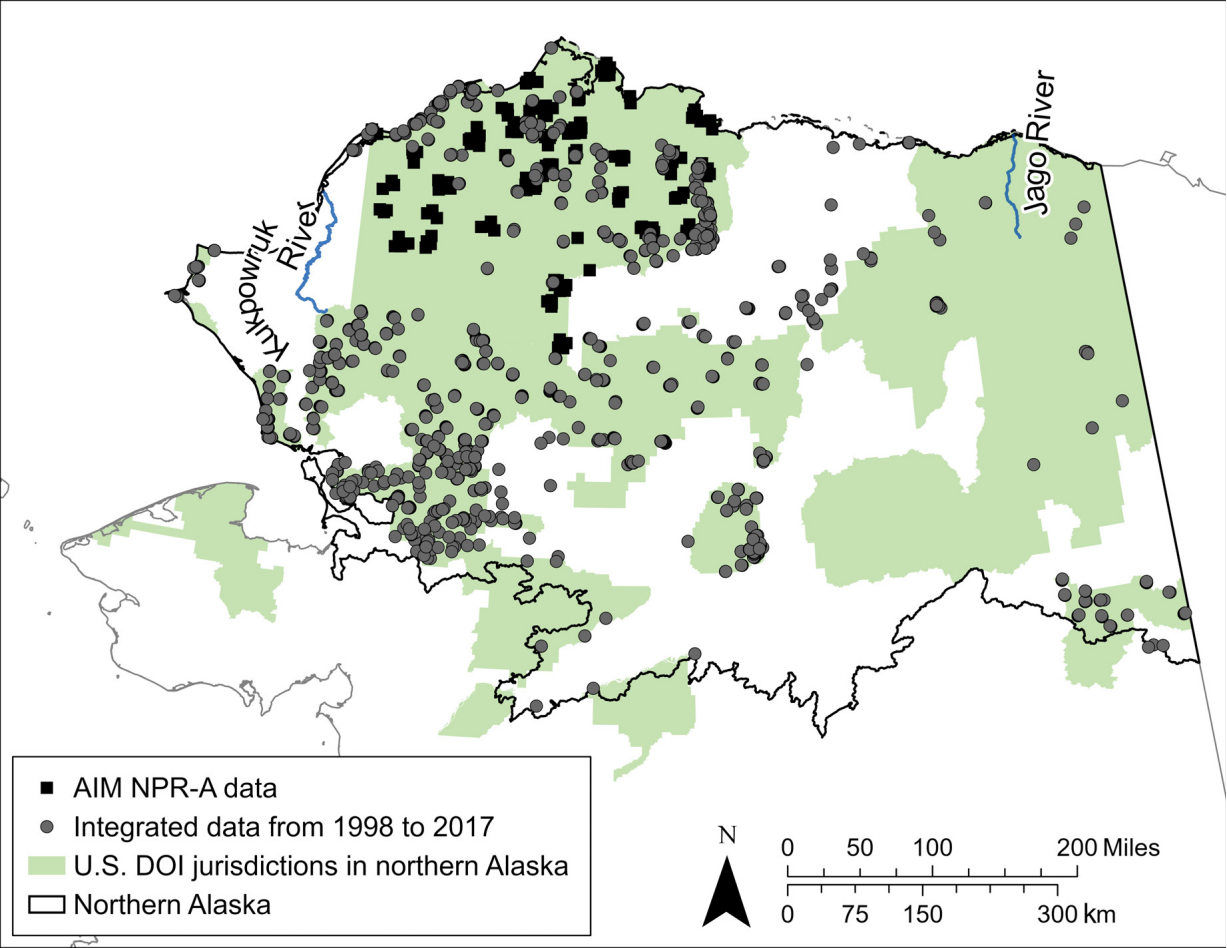
Locations of our vegetation observations (black squares) used to train both the classifiers and regressors, and locations of additional vegetation observations collected from 1998 to 2017 in northern Alaska (gray circles) used only to train the classifiers. Areas in green represent U.S. Department of the Interior (DOI) jurisdictions within northern Alaska.

The physical space within which valid statistical inference can be made depends on the environmental variation represented by the field samples, the selection of covariates to relate to the responses, and the spatial heterogeneity of the landscape. We amassed numerous vegetation observations by integrating multi‐project data into a standardized database for the prediction of species distributions (Fig. 1). However, we selected only our foliar cover observations for the prediction of foliar cover to avoid introducing variation related to inconsistencies in sampling method and plot size. We therefore limited our study area to include only the region where the majority of environmental variation was represented by our sample sites.

We delineated our study area as the largest contiguous region where at least 50% of physical space within a moving 1.5 × 1.5 km grid was within the support vector in multivariate space that bounded 95% of our vegetation observations (Schölkopf et al. 2001). We converted the calculated study area to a smoothed polygon, adjusted the west and east boundaries to follow rivers, and made small manual adjustments to further smooth the polygon. Because an exploratory analysis revealed that observations from a larger spatial extent improved the accuracy of distribution predictions within the final study area, we did not filter the training observations to match the final study area. Instead, the final study area only limited our predictions to the region where our accuracy estimates for the foliar cover maps were valid.

### Continuous foliar cover models

To generate continuous foliar cover maps for each species, we predicted distribution of species using a probabilistic classifier trained on all available integrated observations for northern Alaska. Within the predicted presences, we predicted foliar cover of species using a regressor trained on our consistent and quantitative foliar cover observations. The center point of each site provided the spatial representation of the data. At each site, we rounded foliar cover to the nearest integer percentage per species. Trace cover of dominant or widespread species is often linked to the occurrence of micro‐habitats that cannot be represented by moderate‐scale environmental or spectral covariates. In addition to true absences, we considered observations of the species at less than 0.5% cover, including values of “trace,” to be absences to maintain consistent rounding to the nearest integer percentage. We constructed both the classifiers and the regressors as Bayesian‐optimized stochastic gradient boosting ensembles.

We selected stochastic gradient boosting ensembles, implemented in XGBoost (Chen and Guestrin 2016), as the most appropriate modeling algorithm for predicting spatial variation given that we incorporated collinear variables and suspected numerous weakly informative relationships, important multivariate interactions, and non‐linearities among responses and covariates. Stochastic gradient boosting ensembles combine iterative weak learners, where each weak learner performs slightly better than random, to sequentially fit the gradient remaining from combined previous weak learners to minimize a loss function (Hastie et al. 2009, Kuhn and Johnson 2013, Chen and Guestrin 2016). We selected decision trees as the weak learners in our models because they inherently handle nonlinear relationships, are resistant to outliers, internally select for the most informative covariates, and can be forced to perform as weak learners by limiting the number of splits (Friedman 2002, Hastie et al. 2009). We included random selections of observations and covariates in each iteration and split to prevent overfitting (Friedman 2002, Chen and Guestrin 2016). Maximization of predictive generalizability and prevention of overfitting require tuning model hyperparameters to the individual structure of each data problem (Hastie et al. 2009, Cawley and Talbot 2010, Chen and Guestrin 2016). We optimized eleven hyperparameters for each model in a Bayesian statistical framework using a Gaussian process generative model implemented in GPyOpt (González et al. 2014). The resulting models were thus uniquely tuned and fit to the ecological variation in each species and response.

For each species, we combined predictions of absence from the classifier with predictions of foliar cover percentage from the regressor to form a composite model. The threshold that minimized the absolute value difference between sensitivity and specificity from independent validation partitions determined the conversion of the probabilistic classifier predictions to binary presence and absence (Liu et al. 2005, Jiménez‐Valverde and Lobo 2007). Because of the multi‐step nature of our modeling process, we nested an inner *k*‐fold cross‐validation to optimize hyperparameters and conversion thresholds on independent validation partitions within the training partitions of an outer *k*‐fold cross‐validation. The test partitions of the outer cross‐validation were each used only a single time to evaluate the performance of the composite model. Nested cross‐validation ensured the independence of the test partitions and thus prevented over‐estimation of performance, which can occur when the independence of test partitions is compromised (see Hastie et al. 2009, Cawley and Talbot 2010). Because a *k* value of 10 has been demonstrated as a good compromise between bias and variance (Hastie et al. 2009, Rodriguez et al. 2009), we set *k* equal to 10 for both the inner and outer cross‐validations. Thus, the outer cross‐validation divided our available data into 10 train‐test partitions, and the inner cross‐validation subdivided each outer train partition into 10 train‐validate partitions. While multiple iterations of cross‐validation allow an assessment of the effects of random partitioning on model performance (Rodriguez et al. 2009), our models were too computationally intensive to calculate more than a single iteration of the outer 10‐fold cross validation. We therefore estimated model performance from the merged test partitions of a single iteration of the outer 10‐fold cross‐validation, wherein each observation was predicted exactly once.

To measure overall model performance, we calculated *R* ^2^, mean absolute error (MAE), and root mean squared error (RMSE) from the continuous foliar cover predictions across test partitions of our quantitative vegetation observations. In the context of predictive models, *R* ^2^ is the proportion of variation explained by a simple linear model for observed values as a function of predicted values where the intercept is 0 and the slope coefficient is 1. To provide a better understanding of how well the models represented the distribution of each species, we also calculated area under the receiver operating characteristic curve (AUC) and accuracy from the probabilistic and binary distribution predictions across test partitions of all integrated vegetation observations for northern Alaska. Combined, the overall performance and the performance of the absence class show how well the predictions represent observed vegetation patterns for the modeled species. Final spatial predictions were calculated from classifiers and regressors trained on all available data, using the same inner cross‐validation scheme for optimization of hyperparameters and conversion thresholds as described above, to ensure the best possible models.

### Covariate importance

We assessed the relationship of individual covariates selected into each model with the response by calculating covariate “importance” per model. XGBoost calculated covariate importance from the final classifiers and regressors as the size‐weighted contribution, based on reduction in squared error, of each covariate in determining splits across all decision trees in the model (Kuhn and Johnson 2013). Because each model had a different set of optimized hyperparameters, the numerical importance values were not directly comparable among models. We therefore only report similarities and trends in covariate importance across models rather than numerical comparisons of importance for individual covariates among models.

### Area by vegetation summary

We summarized results by generalized regions of similar environmental and ecological characteristics defined as ecoregions by Nowacki et al. (2001) and as circumarctic bioclimatic zones by Elvebakk (1999). For each species, we calculated vegetation area as the percentage of foliar cover multiplied by the 900‐m^2^ area of each grid cell summed for all cells in the region. Within the study area, the Brooks Foothills ecoregion was analogous to bioclimatic zone E, and the Arctic Coastal Plain ecoregion was analogous to bioclimatic zones C and D (Elvebakk 1999). Within the Arctic Coastal Plain, we compared our foliar cover predictions to the distribution of geomorphic features in a recent tundra landform map (Lara et al. 2018). We also summarized foliar cover for floodplains and stream corridors in the Brooks Foothills.

### Comparison to categorical vegetation maps

To assess the performance of a categorical vegetation map for predicting species‐level patterns, we used our quantitative foliar cover observations to calibrate discrete mean species‐level foliar cover predictions for the 25 map classes in the North Slope Land Cover (NSLC) map, a categorical raster vegetation map with a 30 × 30 m resolution (Ducks Unlimited 2013). We selected *R* ^2^ as the basis for the performance comparison because it was the only metric for the abundance component that was comparable across models. In addition to the NSLC map, we also assessed the performance of a randomly generated categorical raster map with the same spatial resolution and number of classes as the NSLC map. We included the performance of a random distribution of vegetation classes to provide context to the performance of the NSLC map because a minimum *R* ^2^ of zero cannot be assumed when cross‐validating predictive models with independent test data (Hastie et al. 2009, Kuhn and Johnson 2013). For each of the six species, we estimated the discrete mean foliar cover of each vegetation class using ordinary least squares linear regression models with vegetation class as the independent variable and foliar cover as the dependent variable. We estimated the performance of the linear regressors from the merged test partitions of a single iteration of 10‐fold cross‐validation, such that each observation was predicted exactly once. As with our continuous foliar cover maps, we calculated *R* ^2^ across test partitions as the proportion of variation explained by a simple linear model for observed values as a function of predicted values where the intercept is 0 and the slope coefficient is 1. Through this process, we generated an *R* ^2^ representing performance per species for both the NSLC map and the random map. The resulting *R* ^2^ values were comparable to those that we calculated for our continuous foliar cover maps because we estimated performance for discrete mean foliar cover as a function of vegetation class using the same set of observation data and the same cross‐validation framework with the same partitions.

## Results

### Automated study area delineation

**Figure 2:**
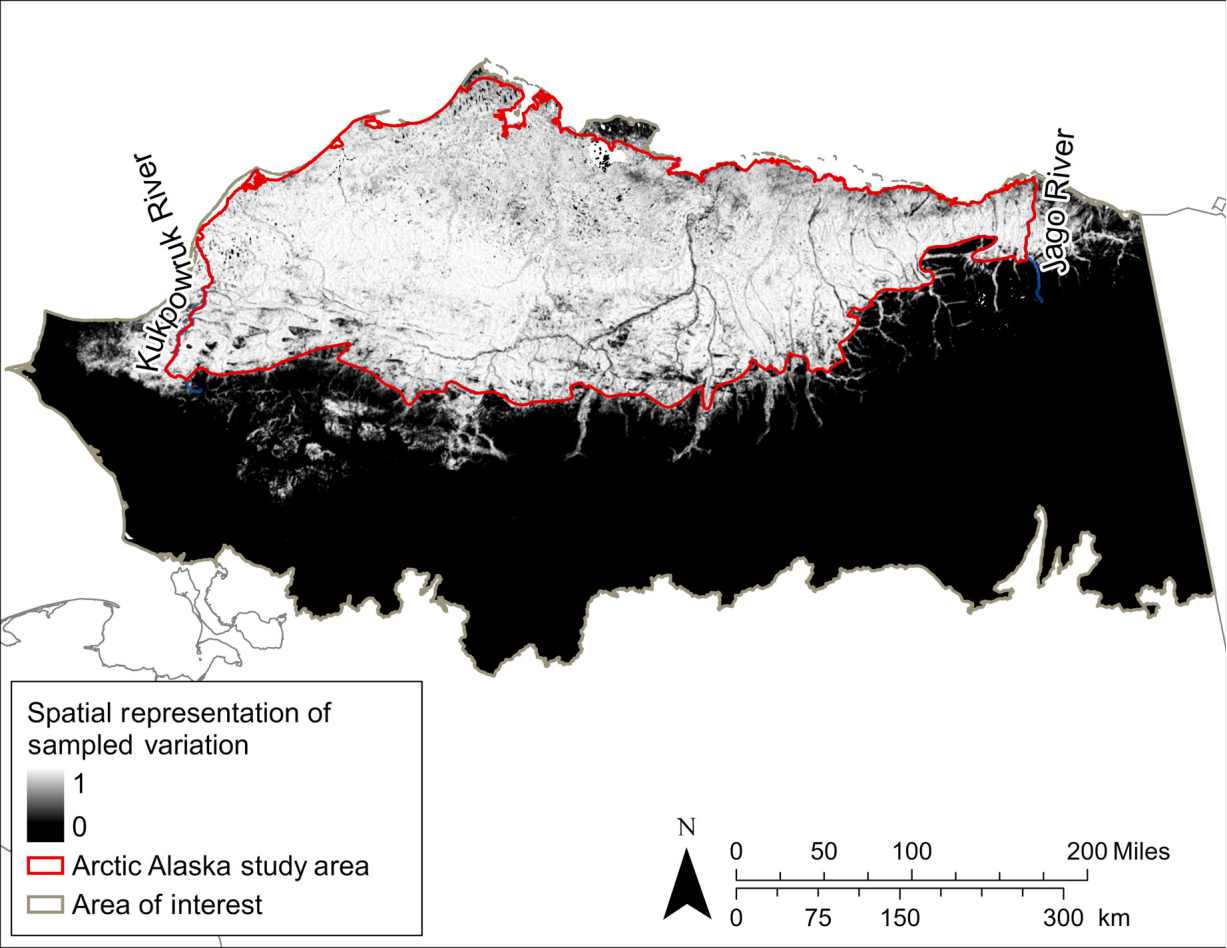
Manually corrected study area compared to the proportion of 30 × 30 m cells within a moving 50 × 50 cell (1.5 × 1.5 km) window that were within the support vector that bounded 95% of our vegetation observations in multivariate space. Black cells within the study area primarily represent rivers, lakes, and unvegetated surfaces, which we did not sample.

The study area included most of the land north of the Brooks Range between the Kukpowruk and Jago rivers (Fig. 2). Except for the more limited western and eastern extent, our study area corresponded approximately to the Arctic Coastal Plain and Brooks Foothills ecoregions as defined by Nowacki et al. (2001). We mapped 140,500 km^2^ of arctic Alaska. Notably, our vegetation observations did not adequately represent the small areas north of Teshekpuk Lake between Smith and Harrison bays and from Utqiagvik to Point Barrow along the Beaufort Sea coast. These unrepresented regions lie within bioclimatic subzone C, which has a limited extent in extreme northernmost Alaska and is ecologically important (Walker et al. 2018). Although we limited sampling to the western half of arctic Alaska, our data covered the environmental variation across much of the Arctic Coastal Plain and Brooks Foothills.

### Foliar cover

Although foliar cover theoretically ranges to 100%, species in arctic Alaska had foliar cover greater than 50% in less than 1% of our observations and greater than 25% in only 4% of our observations. Because we selected species by frequency of occurrence in our observation data, all were widespread in arctic Alaska. Even so, the distributions of foliar cover observations for each species were dominated by zero. Three major patterns in abundance emerged from the mapped species: *C. aquatilis* and *E. vaginatum* were frequently dominant (≥ 25% foliar cover) when present; *S. pulchra* and *E. angustifolium* were dominant only in a narrow set of environmental and biotic conditions; and *R. tomentosum* and *V. vitis‐idaea* were common but rarely dominant.

Performance of continuous foliar cover maps ranged from an *R* ^2^ of 0.65, a map that reflected most of the observed variation, to an *R* ^2^ of −0.06, a map that performed similarly to uniform assignment of mean foliar cover across the landscape (Table 5). Out of the six selected species, only the model for *E. angustifolium* failed to produce satisfactory results. RMSE and MAE were highest for *C. aquatilis* and *E. vaginatum*, the two species that were frequently dominant when present and that therefore had the most potential for large errors in the upper ranges of foliar cover. RMSE and MAE were relatively low for *R. tomentosum*,* S. pulchra*, and *V. vitis‐idaea,* all of which were dominant in restricted landscape settings or were infrequently dominant. The relatively high *R* ^2^ achieved by the model for *E. vaginatum* suggests that the life‐form difference between sedges and shrubs did not play a role in differences in predictive performance for foliar cover. The distribution classifiers for all species, including *E. angustifolium*, performed well, with the lowest AUC being 0.82 for *S. pulchra* and the highest AUC being 0.91 for *E. vaginatum*. Prediction raster data sets of foliar cover, along with trained statistical models and supplementary plots, for the five species with satisfactory performance are available for download online (see *Data Availability*).

**Table 5:** *R* ^2^, MAE, and RMSE per species calculated from the merged test partitions of a single iteration of 10‐fold cross‐validation of our quantitative foliar cover observations, and predictive performance of species absence measured by AUC and percent accuracy calculated from the merged test partitions of a single iteration of 10‐fold cross‐validation of all integrated distribution data for northern Alaska

Species	Overall performance			Presence–absence performance		Mean and standard deviation observed foliar cover (%)
	*R* ^2^	MAE (% cover)	RMSE (% cover)	AUC	Accuracy (%)	
*E. vaginatum*	0.65	6.3	11.3	0.91	83	11.3 ± 19.1
*S. pulchra*	0.62	3.3	6.1	0.82	74	4.7 ± 10.0
*R. tomentosum*	0.61	2.6	4.9	0.89	81	4.8 ± 7.8
*V. vitis‐idaea*	0.56	3.3	5.8	0.89	82	6.0 ± 8.8
*C. aquatilis*	0.36	9.4	14.3	0.87	79	13.3 ± 17.9
*E. angustifolium*	−0.06	6.2	10.0	0.86	79	5.2 ± 9.7

Notes

Mean and standard deviation for observed foliar cover from our quantitative foliar cover observations provide context to MAE and RMSE.

**Figure 3:**
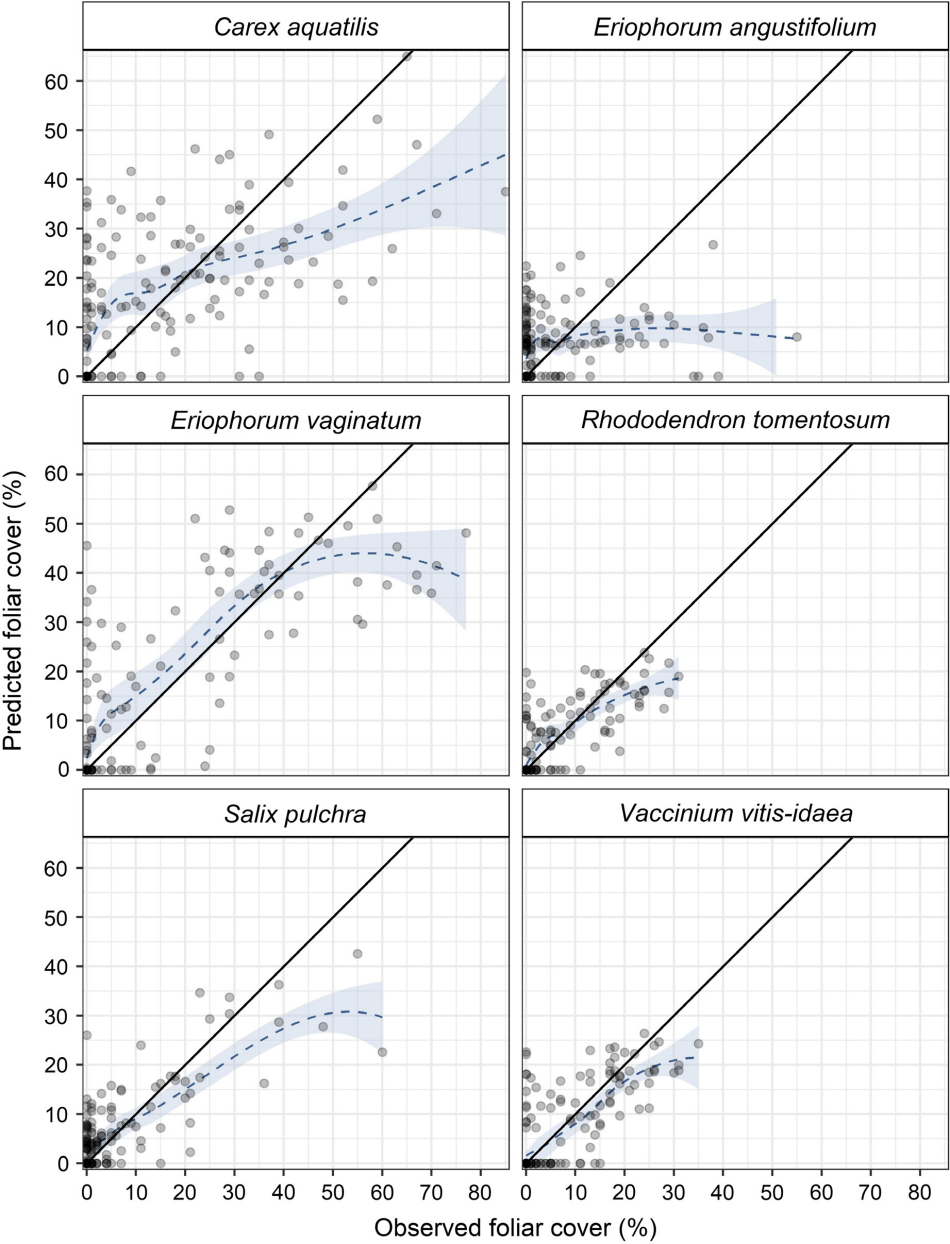
Observed foliar cover compared to predicted foliar cover from the merged test partitions of 10‐fold cross‐validation, wherein each observation was predicted exactly once (semi‐transparent gray circles). *R* ^2^ values were calculated relative to the theoretical 1:1 ratio between observed and predicted foliar cover (solid black line). The loess smoothed conditional means (dark blue dashed curves with 95% confidence intervals in light blue) show the general overpredictions of low observed foliar cover values and the general underpredictions of high observed foliar cover values for all species.

Because of the similar performance of all species classifiers, with mean absence classification accuracy of 80%, the composite models for all species made small but significant overpredictions of zero and tended to overpredict the lower range of observed foliar cover values (Fig. 3). The composite model for *C. aquatilis* significantly overpredicted observed foliar cover values approximately less than 12%. The composite models for each of the shrub species tended to overpredict low observed foliar cover values, up to between approximately 5% and 10%, depending on the species. For *E. vaginatum*, the composite model tended to overpredict observed foliar cover values up to 40%. Additionally, the composite models for all species significantly underpredicted the high observed foliar cover values. For example, the composite model for *E. vaginatum* had decreased ability to distinguish foliar cover values of approximately 50–77%. However, only 7% of our observations of *E. vaginatum* had foliar cover ≥ 50%. Our results showed regional gradients in all mapped species (Table 6), which correspond to broad climatic and moisture gradients north to south across the study area.

**Table 6:** Percentage of total area and total area per species for ecoregions and bioclimatic zones within the study area

Ecoregion or bioclimatic zone	Percentage of total area and area per species				
	*C. aquatilis*	*E. vaginatum*	*R. tomentosum*	*S. pulchra*	*V. vitis‐idaea*
Arctic Coastal Plain	19% (10,137 km^2^)	14% (7,607 km^2^)	5% (2,598 km^2^)	3% (1,639 km^2^)	5% (2,741 km^2^)
Zone C	21% (980 km^2^)	7% (340 km^2^)	0.7% (34 km^2^)	2% (92 km^2^)	1% (67 km^2^)
Zone D	18% (10,905 km^2^)	17% (10,232 km^2^)	6% (3,794 km^2^)	4% (2,646 km^2^)	7% (4,040 km^2^)
Brooks Foothills	8% (6,548 km^2^)	25% (21,568 km^2^)	12% (9,845 km^2^)	11% (9,181 km^2^)	14% (11,610 km^2^)
Zone E	6% (4,795 km^2^)	25% (18,645 km^2^)	12% (8,638 km^2^)	11% (8,104 km^2^)	14% (10,269 km^2^)

Note

The Arctic Coastal Plain ecoregion is analogous to bioclimatic zones C and D, and the Brooks Foothills ecoregion is analogous to zone E.

**Figure 4:**
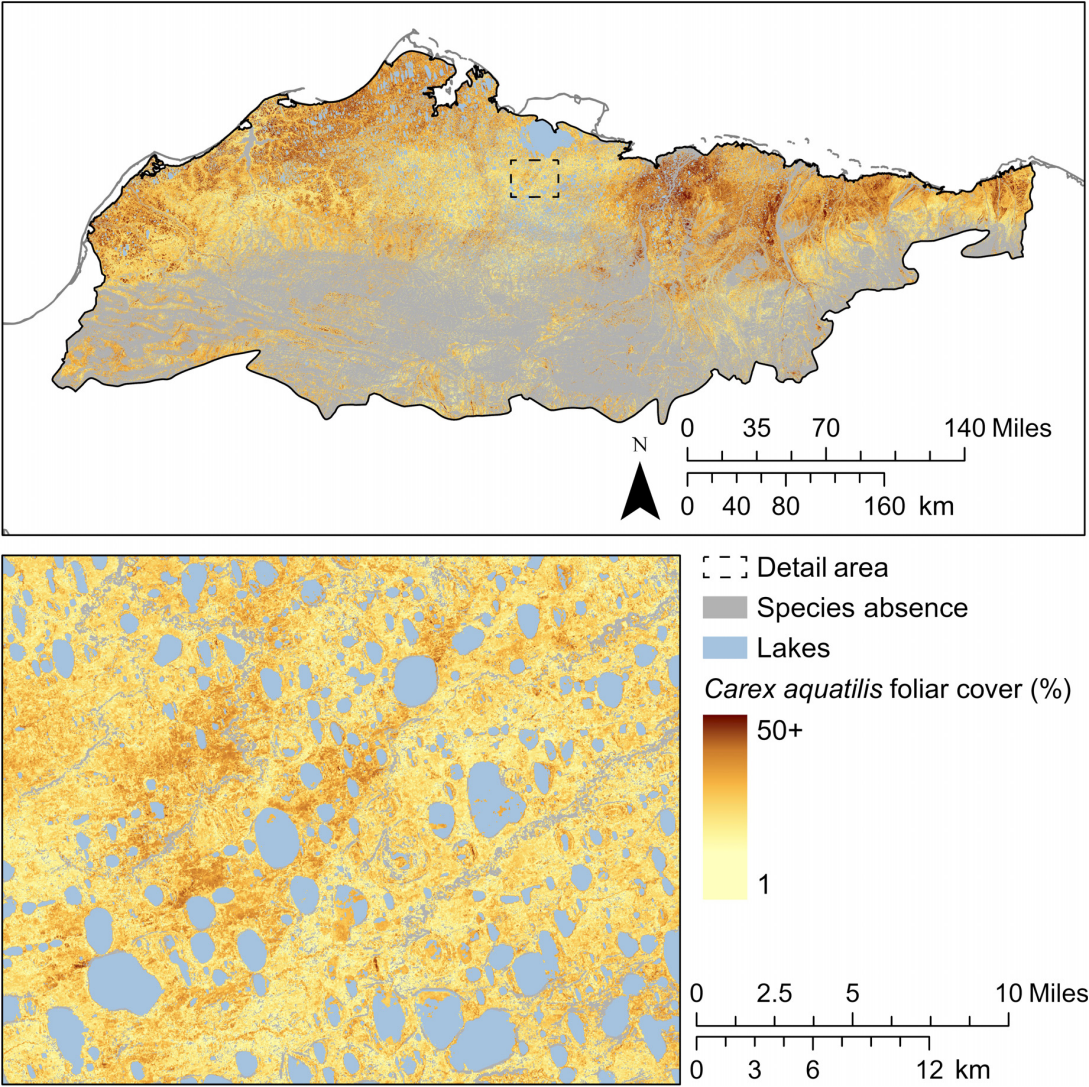
Predicted distribution and continuous foliar cover of *C. aquatilis*.

*Carex aquatilis* was widespread in wetlands. Where the composite model predicted presence of *C. aquatilis*, 95% of predicted foliar cover was between 5% and 46% (Fig. 4). Based on a comparison of our continuous foliar cover maps with the distribution of geomorphic features in a recent tundra landform map (Lara et al. 2018), absences of *C. aquatilis* within the Arctic Coastal Plain were primarily associated with deep standing water and well‐drained gravel and mineral substrates of floodplains and dunes. Of landforms in the Arctic Coastal Plain, *C. aquatilis* had the greatest proportional cover on low‐centered ice wedge polygons (defined in Lara et al. 2018) at 28% (3,244 km^2^). In the Brooks Foothills, *C. aquatilis* was largely absent from the drained slopes that dominate the region but covered 12% (334 km^2^) of small stream corridors.

**Figure 5:**
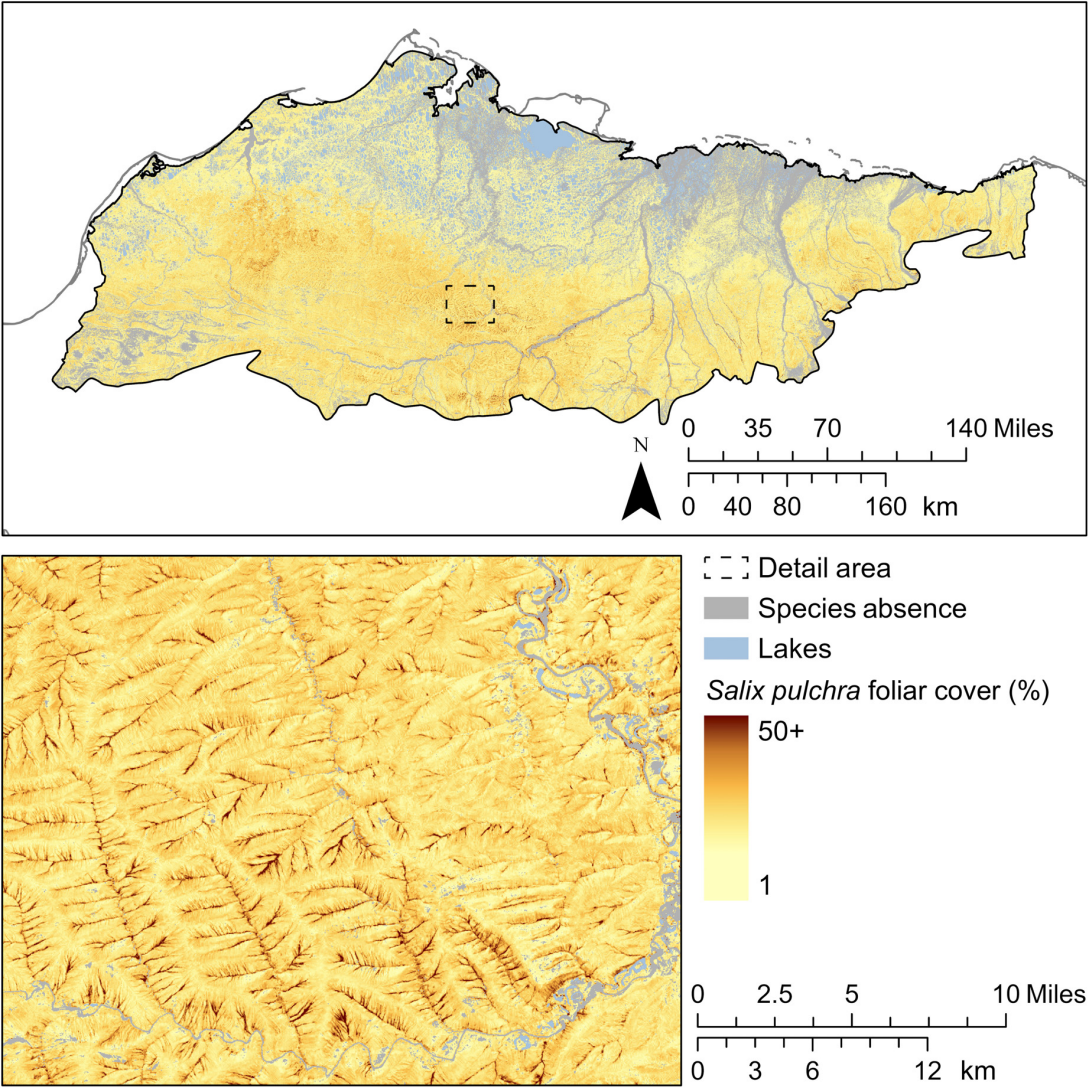
Predicted distribution and continuous foliar cover of *S. pulchra*.

*Salix pulchra* was widespread at low foliar cover except for the wettest areas, where it was absent. Where the composite model predicted presence of *S. pulchra*, 95% of predicted foliar cover was between 2% and 24% (Fig. 5). On the Arctic Coastal Plain, *S. pulchra* had the greatest proportional cover on drained slopes at 7% (298 km^2^) and high‐centered polygons at 4% (773 km^2^). In the Brooks Foothills, *S. pulchra* covered a greater proportion of small stream corridors at 13% (351 km^2^) and a lower proportion of floodplains at 6% (385 km^2^) than the mean foliar cover for the ecoregion (11%).

**Figure 6:**
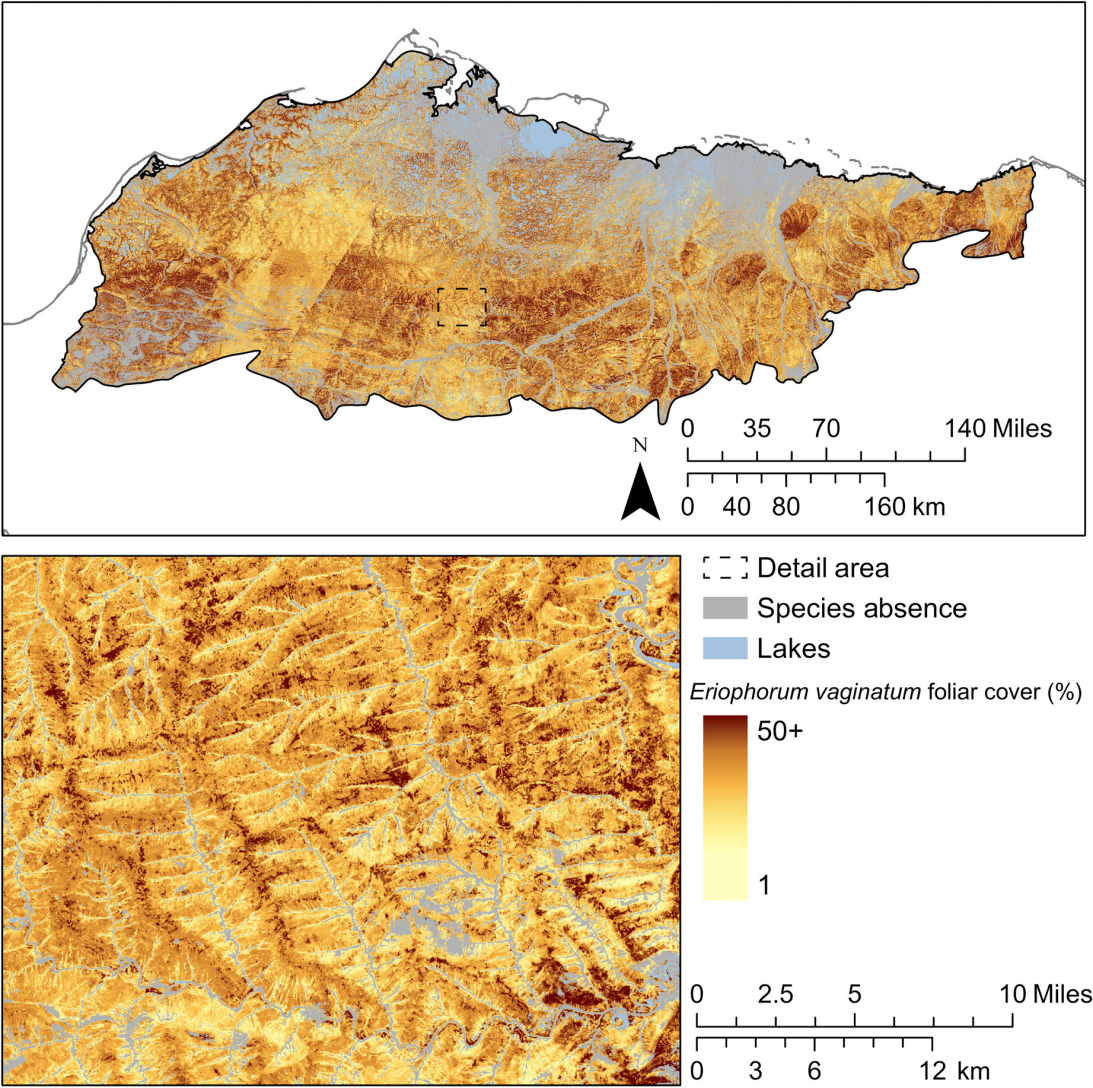
Predicted distribution and continuous foliar cover of *E. vaginatum*.

**Figure 7:**
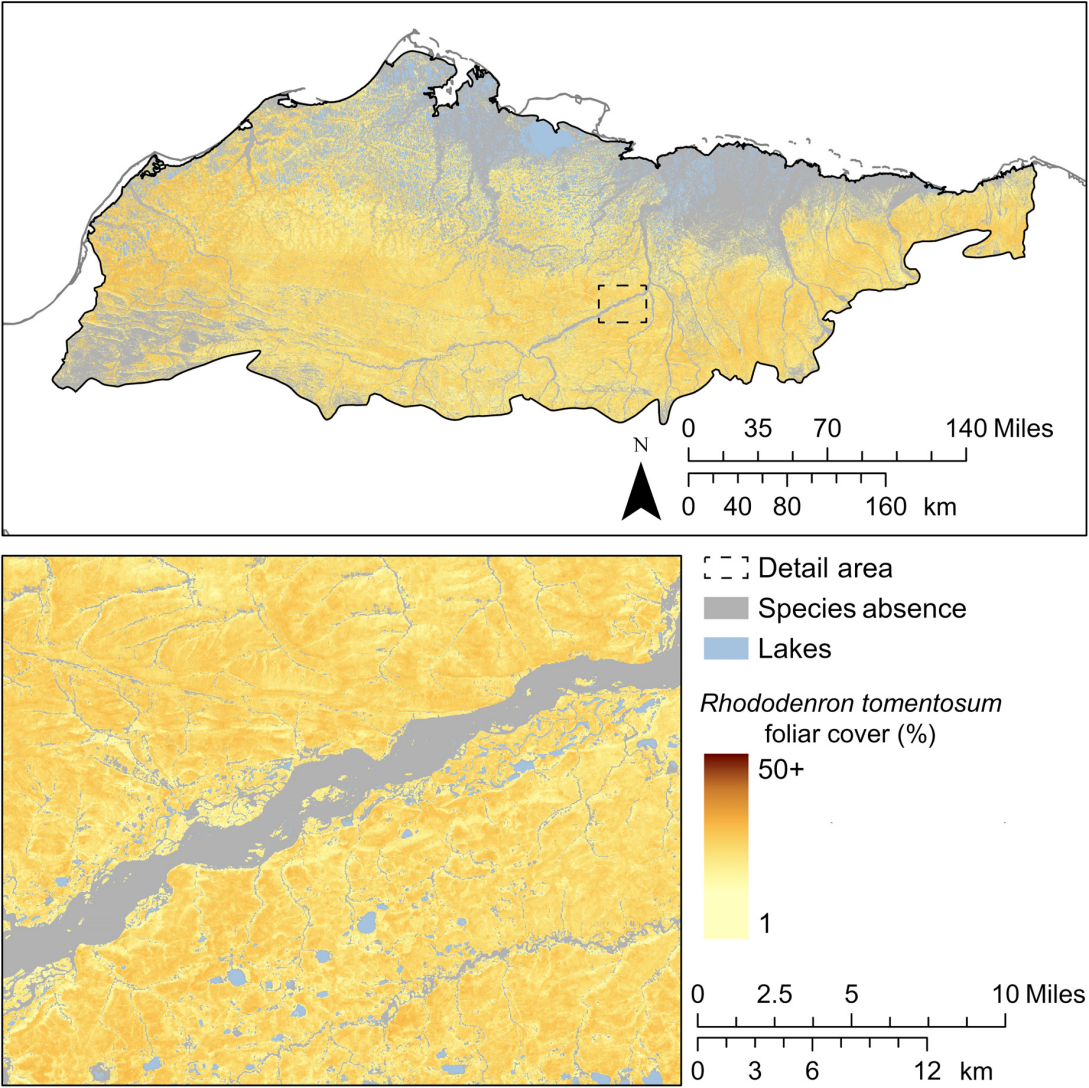
Predicted distribution and continuous foliar cover of *R. tomentosum*.

**Figure 8:**
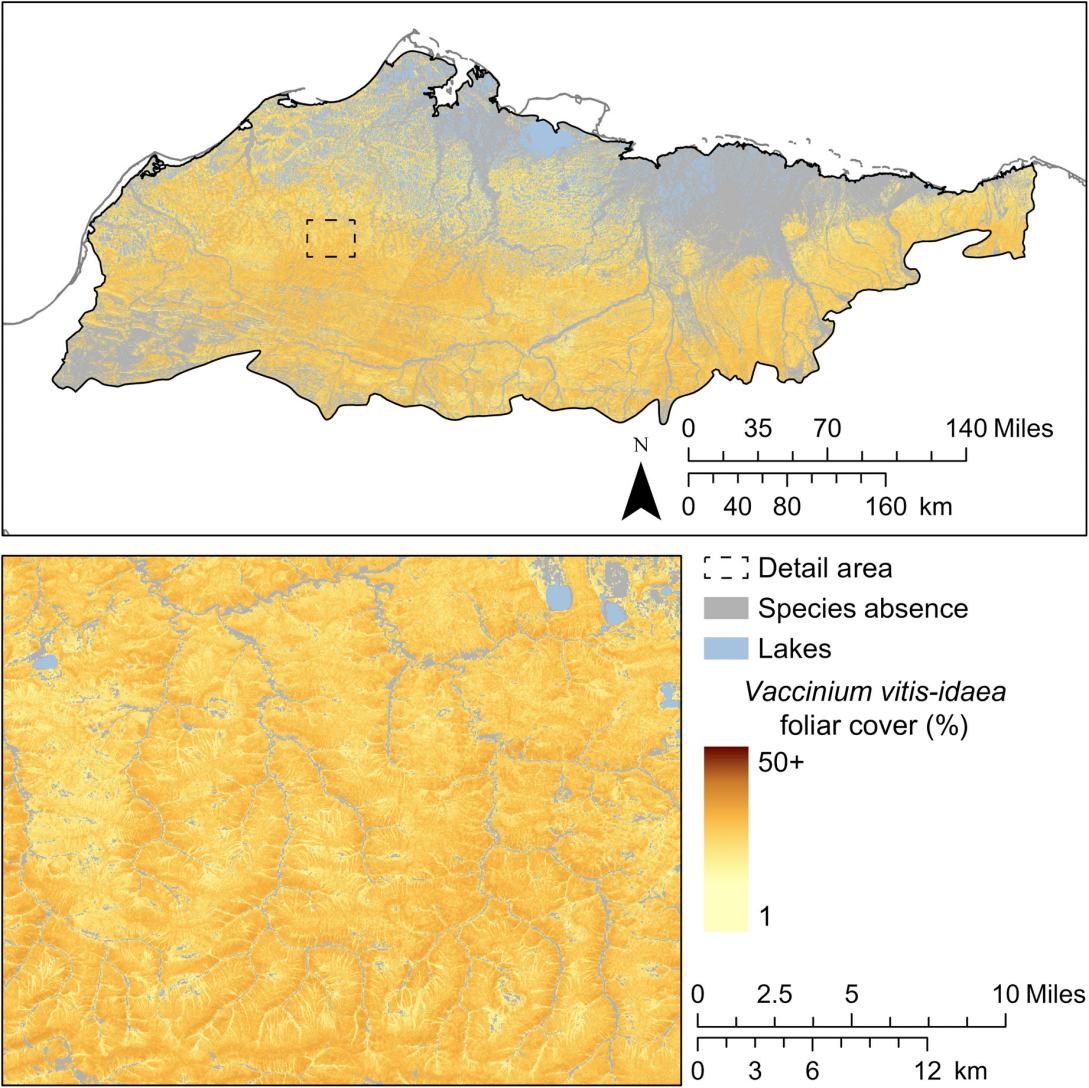
Predicted distribution and continuous foliar cover of *V. vitis‐idaea*.

Correlations in foliar cover among *E. vaginatum*,* R. tomentosum*, and *V. vitis‐idaea* were strong and positive (*r *>* *0.82) for both observations and predictions. Additionally, the strengths of correlations among these species were similar between observations and predictions. *E. vaginatum* was widespread on drained slopes and often dominant (≥ 25% foliar cover). Where the composite model predicted presence of *E. vaginatum*, 95% of predicted foliar cover was between 6% and 53% (Fig. 6). *R. tomentosum* and *V. vitis‐idaea* were also widespread and distributed similarly to *E. vaginatum*. Unlike *E. vaginatum*, each was infrequently dominant with 95% of predicted foliar cover where present between 4% and 22% for *R. tomentosum* (Fig. 7) and between 6% and 26% for *V. vitis‐idaea* (Fig. 8). Of landforms in the Arctic Coastal Plain, *E. vaginatum* had the greatest proportional cover on drained slopes at 29% (1,236 km^2^) and high‐centered polygons at 24% (4,167 km^2^). *R. tomentosum* and *V. vitis‐idaea* also each had the greatest proportional cover on high‐centered polygons at 8% (~1,500 km^2^) and drained slopes at 11% and 12%, respectively (~500 km^2^). These species were largely absent from well‐drained mineral substrates and low‐slope wetland landforms, such as coalescent low‐centered polygons, drained thaw lakes, and lake margins. In the Brooks Foothills, these species were widespread on drained slopes. The foliar cover prediction for *E. vaginatum* was sensitive to a seam in the spectral composites representing different phenological states within a single month.

### Environmental and spectral covariate importance

Several biologically relevant topographic, hydrographic, and climatic covariates were of high importance, determined as being within the ten most important covariates per model, in all classifiers and some regressors. Elevation, aspect, and representations of surface texture were the most consistently important environmental covariates across models. Compound topographic index and integrated moisture index, both of which quantify topographic capacity for moisture accumulation, were of high importance in several models. In contrast to topographic and moisture covariates, hydrographic covariates were generally of low importance and occasionally contributed almost no reduction in squared error. Temperature covariates were of high importance for the classifiers of *C. aquatilis*,* E. angustifolium*, and *S. pulchra*. Although general trends of covariate importance are recognizable, patterns of covariate importance differed both among species and between distribution and abundance responses.

Sensitivity to variation in spectral reflectance was of high importance across all models. Irrespective of month, NDVI and metrics related to water (NDWI and NDMI) were highly important for most models. EVI2, NBR, and NDSI were of high importance for some species–response combinations. Collectively, raw spectral properties were of high importance for most models. Green, Short Infrared 1, and Near Infrared were the most important covariates in the regressors for *C. aquatilis*,* R. tomentosum*, and *V. vitis‐idaea*, respectively. When aggregated by month, May and June (i.e., early season) spectral covariates were most frequently of high importance. Spectral covariates from September were also of high importance in most models. While patterns of importance among spectral properties and months were apparent in our models with aggregation, the importance of individual spectral covariates was generally inconsistent among models because of the inclusion of multiple months per spectral property. We provide the covariate importance histograms with the prediction raster downloads for each species, but we caution users from direct numerical comparisons for individual covariates across models.

### Comparison to categorical vegetation maps

Our continuous foliar cover maps for all species except *E. angustifolium* predicted more of the observed species‐level variation in foliar cover than the North Slope Land Cover (NSLC) map (Table 7). Our continuous foliar cover maps showed an increase over the *R* ^2^ of the NSLC map of 0.12 for *V. vitis‐idaea* at a minimum and 0.54 for *S. pulchra* at a maximum. The distribution of 25 random discrete classes performed worse than assigning the mean foliar cover across the landscape for all species. Therefore, even where *R* ^2^ was close to zero, our continuous foliar cover maps and the NSLC map performed better than a random discrete map. The NSLC map predicted the spatial heterogeneity of *E. vaginatum*,* R. tomentosum*, and *V. vitis‐idaea* best out of the tested species. The proportional improvements of our continuous foliar cover maps over the categorical map were greatest for *C. aquatilis* and *S. pulchra*.

**Table 7:** Comparison of the proportion of observed variation in species‐level foliar cover predicted by a random distribution of 25 map classes, the NSLC Map, and our continuous foliar cover maps

Species	*R* ^2^ of observed species‐level foliar cover predicted			Difference between continuous and NSLC map *R* ^2^
	Random discrete class map	NSLC discrete map	Continuous foliar cover maps	
*S. pulchra*	−0.25	0.08	0.62	0.54
*C. aquatilis*	−0.22	0.15	0.36	0.21
*R. tomentosum*	−0.10	0.43	0.61	0.18
*E. vaginatum*	−0.27	0.49	0.65	0.16
*V. vitis‐idaea*	−0.14	0.44	0.56	0.12
*E. angustifolium*	−0.24	0.00	−0.06	−0.06

## Discussion

We successfully modeled foliar cover of species using a Bayesian‐optimized stochastic gradient boosting ensemble approach for five dominant plant species in Alaska's Arctic Coastal Plain and Brooks Foothills. The diversity of important covariates among models suggest that vegetation patterns at the species level cannot be adequately quantified from a common set of a few universally representative covariates. Comparison of our results to the predictions of a categorical vegetation map show that species‐level continuous foliar cover maps predict more of the observed variation in species distribution and abundance (i.e., better represent vegetation patterns) than categorical maps. We also compare our results to an overlapping example of plant functional type continuous foliar cover maps (Macander et al. 2017) and find that the species‐level maps reveal ecological patterns not apparent in the plant functional type maps. While we focus on arctic Alaska as a regional test case, our mapping approach is based on fundamental ecological theory and therefore generalizable to other systems.

### Spatial sample representativeness

Our approach for statistically defining a study area as a spatial region of highly represented variation relative to a sample avoided a priori assumptions of the structure of variation across the landscape. This method for determining spatial sample representativeness could provide an approach for selecting field sites that are maximally representative of regional environmental variation for ecological studies, which has been identified as an important goal in landscape ecology (Hoffman et al. 2013). Whereas Hoffman et al. (2013) created spatiotemporal clusters of environmental variation primarily from climate data, our method incorporated topographic, hydrographic, climatic, and spectral data to assess sample representativeness. In conjunction with clustering approaches (e.g., Hoffman et al. 2013, Rowland et al. 2016), our approach could be applied to site stratification while developing field study or survey designs to ensure optimal sampling of environmental and biotic variation within a set of spatial and temporal constraints. A clustering and 95% bounded interval approach would remove the bias inherent in stratifying site selection based on subjectively defined units, which do not account for internal structure that may be important for sampling in certain studies.

### Foliar cover

Our statistical methods provided an effective approach for representing vegetation patterns as the realized niches of species measured through the composite of distribution and foliar cover. The Bayesian framework for hyperparameter selection allowed application of a single modeling algorithm across species without requiring a priori assumption of consistent data patterns among species or responses. The differences in selected hyperparameters per model showed that model optimums, and thus the structure of the underlying data, varied both by species and response. Avoidance of a priori assumptions is an advantage of stochastic gradient boosting ensembles (Hastie et al. 2009). Our results show the applicability of Bayesian‐optimized stochastic gradient boosting ensembles for ecological predictions in unmanipulated, nonequilibrium systems. As additional consistent and quantitative foliar cover observation data are collected in the future, larger sample sizes will better quantify the certainty and accuracy of our methods.

The scale of spatial heterogeneity for foliar cover of *E. angustifolium* was poorly matched to the 30 × 30 m resolution of our analysis, likely resulting in the poor performance of the foliar cover predictions despite strong performance from the distribution predictions. *E. angustifolium* tends to be restricted to nearly linear geomorphic features (often 1–2 m in width), including narrow water tracks, edges of ice‐wedge polygons, and thaw pond margins. The distribution predictions for *E. angustifolium* still provide more information on the functional roles of plant species and communities than categorical vegetation maps because categorical maps only indirectly indicate the potential species associations to a particular type. Our results showed that the spatial patterns of heterogeneity were not equally represented by available covariates or at the 30 × 30 m resolution for all species. Greater care must be taken to match the field observation techniques and resolution to the resolution of available environmental and spectral covariates. In general, we suggest that future foliar cover modeling at higher spatial and spectral resolution (e.g., field data collected in 10 × 10 m plots, Sentinel 2 spectral data, and a 10‐m DEM) would improve the model results. It is worth noting that if past vegetation change is of primary interest, then the spatial and band resolution of previous spectral data may be limited.

Temporal variation is important for ecological processes and relationships; however, our predictions represent a generalized point in time. Phenological differences can be notable between the timing of ground observations and the temporal variation introduced by compositing images taken during different years and different times of any given month. This limitation introduced a composite seam artifact into the spatial prediction for *E. vaginatum* (Fig. 6). Foliar cover of deciduous and herbaceous vascular plants fluctuates seasonally, but our predictions were calibrated to the dates of ground observation. For some applications, the predicted foliar cover can only serve as a surrogate for a characteristic driving a process or relationship of interest. For example, caribou in Arctic Alaska strongly select for emerging inflorescences of *E. vaginatum* in late spring (e.g., Russell et al. 1993). The mid‐summer foliar cover of our *E. vaginatum* prediction is relevant for representing the late spring density of emerging inflorescences but does so indirectly.

Although the years of available Landsat 8 imagery (2013–2017) generally matched well with the years of our vegetation sampling (2012–2017), the spectral composites selected pixels from individual years so that the years of ground observation and satellite observation were not necessarily the same. This temporal mismatch was more exaggerated for our distribution data, which included ground observations up to 15 years older than the oldest satellite imagery. Thus, intra‐ and interannual variation and mismatch between timing of ground and remote observations likely contributed to the variation that our models were unable to explain.

### Correlation among species

Based on correlation among species in both the observations and predictions, our results showed three broad ecological patterns in the spatial heterogeneity of species‐level foliar cover. *C. aquatilis* had highest foliar cover in wetlands and was largely absent from areas that lacked wetland landforms. *S. pulchra* was widespread with respect to soil moisture regime, except for the wettest areas, and had highest foliar cover along stream corridors. *E. vaginatum*,* R. tomentosum*, and *V. vitis‐idaea* were widespread on mesic landforms, such as drained slopes, flat‐centered polygons, and high‐centered polygons. The similar spatial patterns predicted individually among *E. vaginatum*,* R. tomentosum*, and *V. vitis‐idaea* were consistent with definitions of *E. vaginatum* tussock tundra communities that dominate mesic acidic soils in arctic Alaska with *R. tomentosum* and *V. vitis‐idaea* as commonly associated species (Viereck et al. 1992, Raynolds et al. 2008, Walker et al. 2018). The similarity of correlations among species between observations and predictions indicates that our composite models successfully predicted multi‐species patterns. Although we did not include enough species in this test case to fully map plant community composition, our continuous foliar cover maps do partially address plant community composition through the spatial overlap of predicted distribution and abundance. Additional work is required to determine the feasibility of mapping plant community composition as the overlapping spatial patterns of constituent species.

### Environmental and spectral relationships to foliar cover

Contrary to other systems (e.g., Evans and Cushman 2009), a representation of temperature regime was not consistently of high importance, determined as being within the ten most important covariates per model, to predictions of distributions for all selected species. Our study area harbors a range of climatic conditions, with coastal areas under strong maritime influence and central areas dominated by a more continental climate (Young 1971). In the Arctic, summer warmth is associated with the distributional limits of many vascular plants (Young 1971, Walker 2000, Raynolds et al. 2008). However, our species distribution observations did not adequately represent environmental variation in Circumarctic Subzone C, where the distributional limits imposed by summer warmth may be most important for some species (Walker et al. 2005). Additionally, the broad 771‐m resolution of our climate data, which were generated from few and widely spaced weather stations, makes them unsuitable representations of microclimate. The inclusion of both spectral and climate covariates may weaken relationships to the climate covariates where the influences of climate can be inferred indirectly from the imagery.

Moisture gradients are dramatic on the Arctic Coastal Plain where permafrost impedes surface water drainage, and the moisture gradients have a striking impact on the occurrence and abundance of plant species (Walker 2000, Raynolds et al. 2008). Covariates related to moisture (NDWI, NDMI, compound topographic index, and integrated moisture index) were consistently of high importance in our models, reflecting the important influence of moisture gradients on species‐level (and, by extension, community‐level) vegetation patterns at the landscape scale. Higher resolution representations of moisture gradients are likely to improve model performance. Hydrographic covariates were consistently unimportant. We speculate that our DEM was too coarse to provide an accurate representation of streams relative to our mapping resolution and spectral covariates better represented relevant hydrographic properties. Because stream networks are computationally intensive to calculate and contributed little to our inference of species‐level vegetation patterns, we suggest that they are unnecessary for vegetation mapping efforts. In contrast, the frequent importance of at least some topographic, moisture, and temperature covariates indicate that these covariates should be retained in future vegetation mapping efforts.

Among the spectral covariates, we found that both the raw TOA reflectance bands and the calculated metrics were frequently of high importance. Researchers often regard NDVI as the most relevant spectral metric for inferring vegetation patterns in the Arctic (e.g., Walker et al. 2003, 2005, Laidler et al. 2008, Raynolds et al. 2008, Pattison et al. 2015). While we found support for the consistent importance of NDVI for species‐level vegetation patterns, NDVI was not the most important covariate in any model. Thus, similar to the results of Johnson et al. (2018), our results suggest that NDVI alone is not predictive of species‐ or community‐level vegetation patterns. Early and late growing season reflectances are particularly important for distinguishing patterns of some species. For example, NDMI and NDVI in both June and September were highly important in the classifier and regressor for *S. pulchra*, respectively. Although it is unclear what the biologically relevant shoulder season spectral data are reflecting, our results emphasize the importance of early and late season phenomena in arctic plants (see Ernakovich et al. 2014, Blume‐Werry et al. 2016). Analyses of vegetation patterns benefit from the inclusion of a wide array of spectral metrics and raw bands representing multiple phenological states across the growing season in addition to biologically relevant representations of topography, moisture, and climate. Therefore, when modeling vegetation patterns, ecologists must carefully select statistical frameworks, such as Bayesian and statistical learning methods, that are appropriate to identifying patterns from numerous, often collinear, variables.

### Comparison of species and plant functional type patterns

On a per‐species basis, our species‐level continuous foliar cover maps performed better than, similarly to, and worse than the plant functional type continuous foliar cover maps developed by Macander et al. (2017), where plant functional types were defined by broad growth forms. Our single iteration of 10‐fold cross‐validation produced estimates of model performance most comparable to the Random Forest bootstrap performance estimates of Macander et al. (2017). We therefore reference the bootstrap performance estimates from Macander et al. (2017) for comparisons to our results. Our continuous foliar cover map for *S. pulchra* explained a comparable proportion of observed variation (*R* ^2^ = 0.62) as the low deciduous shrub map (*R* ^2^ = 0.62) of Macander et al. (2017). Compared to the sedge continuous foliar cover map (*R* ^2^ = 0.38) in Macander et al. (2017), our map for *E. vaginatum* performed much better (*R* ^2^ = 0.65) while our map for *E. angustifolium* performed much worse (*R* ^2^ = −0.06). Our map for *C. aquatilis* (*R* ^2^ = 0.36) performed similarly to the sedge map. Comparison of our results with those of Macander et al. (2017) suggest that there is no inherent increase in model accuracy associated with decreased taxonomic and ecological specificity when modeling vegetation patterns.

Continuous foliar cover maps of species illustrate distinct patterns that are obscured by aggregation to plant functional types defined by growth form. For example, the sedge plant functional type in Macander et al. (2017: Supplementary Materials Fig. S1a) was relatively evenly distributed across western arctic Alaska from wetlands to well‐drained sites with absences only in deep water. However, at the species level, differentiation in occupation of the landscape was apparent between two regionally dominant sedges, *C. aquatilis* and *E. vaginatum* (Figs. 4 and 6), in a pattern that followed moisture regime. Comparison of both observed and predicted foliar cover between these sedge species showed weak (*r *=* *−0.18) negative correlations. This example contradicts the frequent assumption (e.g., Ustin and Gamon 2010) that plant functional types represent ecologically meaningful or similar responses of multiple species.

Plant functional types defined as generalist categories, such as growth form or family, aggregate contrary responses of species and smooth existing, potentially important, ecological variation. While generalist plant functional types, such as growth form or family, may be valuable for inference of biophysical processes and vegetation structure, they obscure patterns in ecophysiological diversity, interspecific relationships, and plant community composition (Kattge et al. 2011, Scheiter et al. 2013). It is possible to aggregate modeled results for species into functional types post hoc but impossible to disaggregate modeled results for plant functional types to provide inference of species or communities (Scheiter et al. 2013). Although species‐level maps are of distinct theoretical advantage, our results for *E. angustifolium* illustrate that abundance cannot be successfully mapped for all species individually with existing data. An alternative mapping approach in cases of poor species‐level performance would be narrowly defining plant functional types (as species aggregates) relative to key environmental gradients, such as obligate wetland sedges, rather than by generalist categories (see Bogan et al. 2019). We suggest that a good approach may be to map individual dominant and widespread species where acceptable results can be achieved and ecologically defined plant functional types generally.

### Implications for categorical vegetation maps

The better performance of our species‐level continuous foliar cover maps over the North Slope Land Cover (NSLC) map demonstrates the potential for the niche‐based gradient mapping paradigm to better represent ecologically meaningful vegetation patterns than the categorical mapping paradigm. Our method for assessing the performance of categorical vegetation maps simultaneously evaluated how well the predicted classes matched the training labels and how well the class definitions represented the ecological variation. The mapped classes in the NSLC map were subjectively derived, and therefore human interpretation biased the map to prioritize particular species. For example, the NSLC map poorly predicted the observed variation in *S. pulchra* foliar cover, despite our finding that the majority of the spatial heterogeneity of *S. pulchra* was indeed mappable. The performance of our continuous foliar cover maps did not reflect subjective bias in the definition of mapped units beyond the theoretical subjective bias inherent in defining species. Our results agree with those of Cushman et al. (2010a), who found that the predictive performance of categorical vegetation maps varied greatly among species and that continuous representations of species habitat consistently outperformed categorical representations.

The categorical data model for vegetation mapping limits the ability to model ecological interactions and landscape change (Evans and Cushman 2009, Cushman et al. 2010c, Feilhauer et al. 2011, Coops and Wulder 2019). Our approach to mapping continuous foliar cover for individual species addresses this issue. Species‐level mapping may be applicable to other widespread and common species that, when mapped collectively, could contribute to a better understanding of the spatial patterns in plant community composition. However, further work is needed to determine how well our methods perform for species less widespread than the six that we selected as test cases. Foliar cover observations are zero‐inflated at the species level across plots. We speculate that the largest obstacle to mapping less widespread species will be the low total number of presence observations rather than the low proportion of presence observations because of our hierarchical modeling approach. Successful mapping of less common species, as well as improved mapping of the species we selected, will require a greater total number of observation sites. Development of methods using remote sensing data to reconcile differences in field observation methods, sampling resolution, and plot size are critical to more effectively integrating existing data in future efforts. Large, consistent field observation data sets, which in remote areas may require new survey approaches that mitigate costs, are of primary importance to testing the applicability of our methods to less common species.

### Implications for management

Integration of ground‐based measurements with remotely sensed data is a potentially cost‐effective and efficient approach to facilitating monitoring efforts and understanding diverse landscapes in support of scientifically informed natural resource management decisions (Toevs et al. 2011). Our species‐level foliar cover mapping approach addresses the focal priorities of U.S. Federal vegetation monitoring initiatives, which emphasize the need to quantitatively and spatially describe current species‐ and community‐level vegetation characteristics. Quantitative spatial representations enable future measurement of vegetation responses to climate change, anthropogenic development, and management actions. Several key technologies and new paradigms in statistical learning, cloud computing, remote sensing, and landscape mapping have emerged in recent years that fundamentally alter the ecological scale at which vegetation patterns can be mapped. Integrated data, new technologies, and more ecologically relevant theoretical paradigms are critical to producing spatial representations of vegetation pattern that better reflect observed variation. The continuous foliar cover maps of these five widespread vascular plant species in arctic Alaska provide more detailed, reliable, and useful information to land managers and ecologists than existing categorical vegetation maps. Additionally, the vegetation plots database and script repository that we provide from this study will enable further development of a gradient alternative to categorical vegetation mapping in Alaska and beyond.

## Data Availability

Code for all analyses referenced in this study is available in Zenodo: https://doi.org/10.5281/zenodo.3553109. The standardized vegetation plots database containing integrated vegetation observations from 14 survey and monitoring projects conducted in Northern Alaska from 1998 to 2017 is available in Zenodo: https://doi.org/10.5281/zenodo.2590671. Prediction raster data sets of foliar cover, along with trained statistical models and supplementary plots, for the five species with satisfactory performance are available from the Knowledge Network for Biocomplexity at https://doi.org/10.5063/f1zw1j8p.
